# Generation of a homozygous mutant drug transporter (ABCB1) knockout line in the sea urchin *Lytechinus pictus*

**DOI:** 10.1242/dev.200644

**Published:** 2022-06-06

**Authors:** Himanshu Vyas, Catherine S. Schrankel, Jose A. Espinoza, Kasey L. Mitchell, Katherine T. Nesbit, Elliot Jackson, Nathan Chang, Yoon Lee, Jacob Warner, Adam Reitzel, Deirdre C. Lyons, Amro Hamdoun

**Affiliations:** 1 Center for Marine Biotechnology and Biomedicine, Scripps Institution of Oceanography, University of California San Diego, La Jolla, CA 92093-0202, USA; 2 Department of Biology and Marine Biology, University of North Carolina Wilmington, Wilmington, NC 28403-5915, USA; 3 Department of Biological Sciences, University of North Carolina Charlotte, Charlotte, NC 28223-0001, USA

**Keywords:** Sea urchin, ABC transporter, ABCB1, CRISPR/Cas9, Mutant line

## Abstract

Sea urchins are premier model organisms for the study of early development. However, the lengthy generation times of commonly used species have precluded application of stable genetic approaches. Here, we use the painted sea urchin *Lytechinus pictus* to address this limitation and to generate a homozygous mutant sea urchin line. *L. pictus* has one of the shortest generation times of any currently used sea urchin. We leveraged this advantage to generate a knockout mutant of the sea urchin homolog of the drug transporter ABCB1, a major player in xenobiotic disposition for all animals. Using CRISPR/Cas9, we generated large fragment deletions of ABCB1 and used these readily detected deletions to rapidly genotype and breed mutant animals to homozygosity in the *F*_2_ generation. The knockout larvae are produced according to expected Mendelian distribution, exhibit reduced xenobiotic efflux activity and can be grown to maturity. This study represents a major step towards more sophisticated genetic manipulation of the sea urchin and the establishment of reproducible sea urchin animal resources.

## Abstract (precis)

**Summary:** The utility of *Lytechinus pictus* as a much needed genetically enabled sea urchin resource is achieved through the generation of a homozygous mutant knockout of the drug transporter ABCB1/P-glycoprotein.

## INTRODUCTION

For more than one century, sea urchins have been used in experimental embryology to reveal the mechanisms of development. The biological advantages of this organism include their phylogenetic position as basal deuterostomes, the abundance of eggs and sperm produced, the synchrony and transparency of their embryos, and the ease of zygotic microinjection. Nonetheless, there has been one crucial limitation in this model: the lack of stable genetically modified lines.

Production of urchin lines has not been feasible because of the long generation time of widely used sea urchin species, such as *Strongylocentrotus purpuratus* (Leahy, 1986). Although urchin species with shorter generation times, such as *Lytechinus pictus*, were described long ago (Hinegardner, 1969), the molecular tools to capitalize on their fast growth did not exist. The advent of CRISPR/Cas9 gene editing has reinvigorated interest in a genetic manipulation of a wide range of animals (Doudna and Charpentier, 2014; Matthews and Vosshall, 2020). In sea urchins, CRISPR/Cas9 has been primarily used for direct perturbation of genes in the *F*_0_ generation (Lin and Su, 2016; Liu et al., 2019; Fleming et al., 2021; Wessel et al., 2021). However, the application of CRISPR/Cas9 to stable genetic modification of sea urchins is limited. Only one recent study demonstrated its feasibility, by creating homozygous pigmentation mutants of *Temnopleurus reevesii* (Yaguchi et al., 2020). However, this species is not widely available.

The painted sea urchin *Lytechinus pictus* represents an ideal candidate for the establishment of a genetically enabled sea urchin. Most notably, *L. pictus* has a relatively short generation time (4-6 months), that enables breeding in captivity (Hinegardner, 1969; Nesbit et al., 2019; Nesbit and Hamdoun, 2020), and a recently published genome (Warner et al., 2021), which together open the door to targeted stable mutagenesis. The contributions made using this species range from the seminal discovery of cyclins (Evans et al., 1983) to the first characterizations of echinoderm *cis*-regulatory elements (Xiang et al., 1991), the cytoskeletal controls of cell division (Pal et al., 2022) and axis formation (Henson et al., 2021), and modeling embryonic adaptations against ocean pollution and acidification (Cserjesi et al., 1992; Wu et al., 2015; Smith et al., 2019).

Here, we report the generation of homozygous mutant *L. pictus* lines, using standard culturing and mutagenesis methods that can be reproduced in most sea urchin labs. Our target was the sea urchin homolog of the P-glycoprotein human drug transporter (P-gp/ABCB1). ABCB1 is one of the best studied drug transporters and is well known for its role in xenobiotic metabolism in humans (Szakács et al., 2006; Hamdoun and Epel, 2007; Chufan et al., 2015). It is one of the major rate-limiting determinants of drug penetration at the blood-brain barrier (Schinkel et al., 1994, 1996) and of elimination of dietary and bacterial toxins in the gut (Panwala et al., 1998; Leslie et al., 2005; Cario, 2017), and is a major contributor to drug resistance in pathological states (Robey et al., 2018). ABCB1 also plays a crucial role in determining embryonic susceptibility to environmental contaminants and drugs, whether encountered *in utero* in mammals (Han et al., 2018) or externally by orphan embryos in the marine environment (Hamdoun and Epel, 2007). However, robust knockout animal lines of this gene are limited to mice (Schinkel et al., 1994, 1996). Additional transporter animal models would enable the study of the functions of this important gene in different contexts, such as embryonic development.

In this study, we have generated large deletion mutants of *Lp*-*ABCB1* and bred the mutant animals through to homozygosity in the *F*_2_ generation. The results lay the groundwork for a sea change in the scope and methodology of sea urchin developmental biology research, by the use of this new animal resource. The work also establishes a new animal model of ABCB1 that can be used to reproducibly study the function of this gene in the early embryo.

## RESULTS AND DISCUSSION

### Identification and validation of *Lp-ABCB1* as a CRISPR target

**Fig. 1.**
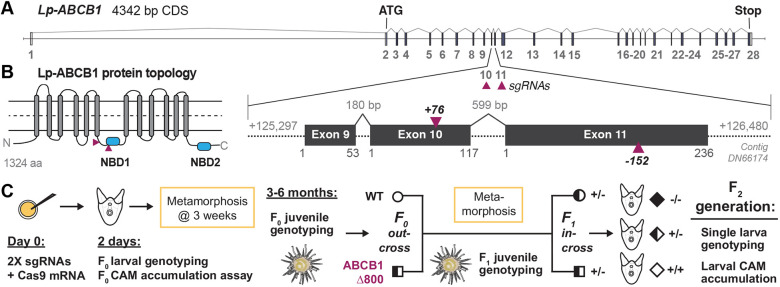
**Generation of a homozygous mutant drug transporter ABCB1^−/−^ line in the sea urchin *Lytechinus pictus*.** (A) The gene locus of *Lp-ABCB1*. The sgRNA target sites (maroon triangles) for generating large deletions between exons 10 and 11 are indicated. (B) Predicted Lp-ABCB1 protein topology. Target site regions in the nucleotide binding domains (NBDs, blue ovals) are shown. NBDs are necessary for transport. (C) Schematic of CRISPR/Cas9 gene editing and propagation of *Lp-ABCB1* mutant generations. Large deletions are generated in the *F*_0_ generation. An *F*_0×_wild-type outcross creates a heterozygous ABCB1 *F*_1_ generation, and the *F*_1_ in-cross generates homozygous mutants in the *F*_2_ generation. Phenotype analysis uses a calcein-AM (CAM) substrate accumulation assay to quantify the level of ABCB1 transporter activity in each generation.

To generate mutants of ABCB1 in *L. pictus*, we first cloned, annotated and validated the gene and protein (Fig. S1; Table S1). The *Lp-ABCB1* locus covers ∼150 kb of sequence across 28 exons (Fig. 1A). Consistent with other known ABCB1 genes, *Lp-ABCB1* encodes a single open reading frame with two membrane-spanning domains, each containing six transmembrane helices and two nucleotide binding domains (Fig. 1B). To validate the subcellular localization and functionality of Lp-ABCB1 protein, an *Lp-ABCB1:mCherry* fusion was generated and overexpressed in embryos (Fig. S2A-C), as previously described (Gökirmak et al., 2012, 2014, 2016). Consistent with the known localization of ABCB1 in other species (Gökirmak et al., 2012), this fusion protein localized to the apical plasma membrane (Fig. S2C). It also effluxed calcein-AM (CAM), a canonical fluorescent substrate of ABCB1 (Fleming et al., 2021; Gökirmak et al., 2012). Embryos overexpressing *Lp-ABCB1:mCherry* accumulated, on average, 38.9% less intracellular calcein when compared with wild-type and *H2B:NmCherry* controls (Fig. S2C,D; *P*<0.0001).

Movement of substrates across membranes by ABC transporters is facilitated by ATP hydrolysis at the nucleotide binding domains (NBDs) of these proteins. Thus, to create loss-of-function mutations in *Lp-ABCB1*, we designed guides uniquely targeting the coding region of the first NBD using CRISPR/Cas9 (Fig. 1A,B; Table S2). The use of dual guides targeting a single gene increases the overall mutation efficiency and can generate large fragment deletions that can be assayed by routine PCR (Zhou et al., 2014; Shin et al., 2017; Eleveld et al., 2021). We targeted two sites: one at exon 10 (Ex10+76) and one at exon 11 (Ex11-152). Genotyping of injected *F*_0_ larvae and settled *F*_0_ juveniles revealed a mix of small indels at each exon and large deletions (up to 800 bp) spanning across both exons (Fig. S3A,B). To confirm that this strategy also perturbs Lp-ABCB1 transporter activity in embryos, a CAM efflux assay was performed, as previously established for the *F*_0_ mutagenesis of ABCB1 in the purple sea urchin (Fleming et al., 2021). *Lp-ABCB1 F*_0_ crispants exhibited a 64% increase in the accumulation of intracellular CAM when compared with controls (Fig. S3C; *P*=7.742E-06).

### Characterization of somatic and germline mutations in *F*_0_ ABCB1 crispants and the selection of founder animals

Having validated a loss-of-function ABCB1 mutation, we next sought to identify founders for breeding a stable mutant line. A total of 19 *F*_0_ crispant urchins were raised through metamorphosis, grown to 3 mm test diameter, and genotyped for somatic and germline mutations. To non-lethally determine somatic mutations, we clipped two or three tube feet from each of the animals for genotyping (Fig. S4A). In these 19 juveniles, 13 had somatic mutations at either or both target sites, including several large deletions (Table S3). Of these 13 individuals, 12 had both frameshift and non-frameshift mutations, and one exhibited only non-frameshift mutations. Six individuals with frameshift mutations were spawned, five of them male (numbers 3-6 and 9) and one female (number 8). All exhibited mutations in their gametes (Fig. S4B). However, not surprisingly given *F*_0_ mosaicism, nearly all of the germline mutations identified were different from the somatic mutations observed in tube feet samples (Fig. S4B,C), except for one animal that retained the same mutation in both tissues (number 5). In addition, two out of the six animals had more than one mutant allele observed in gamete samples, suggesting heterogeneity of mutations in the germline. None of the individuals that were somatic wild type had germline mutations (Fig. S4B,C).

**Fig. 2.**
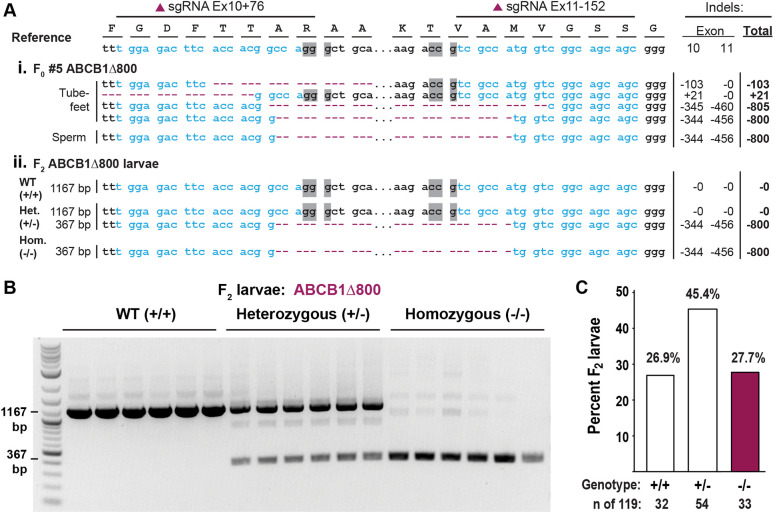
**Identification and propagation of an ABCB1Δ800 bp deletion germline mutant.** (A) Crispant sequence analysis. Genomic DNA was extracted, amplified and cloned from somatic tissue (tube feet) and gametes from metamorphosed juveniles of the *F*_0_ (i) generation, and from individual *F*_2_ larvae (ii). Blue text, sgRNA target sites; gray boxes, PAM site; magenta dashes, indels. Total sizes of indels are indicated for each exon. (B,C) PCR screening of individual *F*_2_ ABCB1Δ800 larvae. Wild type (+/+), and heterozygous (+/−) and homozygous (−/−) mutants were identified by gel band pattern (B). Genotypes from individual *F*_2_ ABCB1Δ800 larvae (*n*=119) demonstrate near Mendelian inheritance of the mutant alleles (C).

Large germline deletions were detected in two *F*_0_ males (numbers 5 and 9). Animal 5 contained only one type of mutation in its sperm: a large 800 bp deletion (hereafter referred to as ABCB1Δ800). This deletion removed the entire intronic genomic sequence between exons 10 and 11, in addition to the exon sequence around both target sites (Fig. 2Ai; Fig. S4B). Importantly, for subsequent screening, ABCB1Δ800 mutants could be readily detected by PCR of the target region as two distinct bands: a 1184 bp wild-type band and 384 bp mutant band (Fig. S4D).

### Generation and genotyping of *F*_1_ and *F*_2_ ABCB1Δ800 mutants

We next bred mutant founders to homozygosity (Fig. 1C; Fig. 2Aii). Sperm from *F*_0_ number 5 were used to fertilize wild-type eggs from two different females and both cultures were raised to produce an *F***_1_** generation, heterozygous for the large deletion mutation (Fig. 1C). A total of 350 juveniles from these batches were produced, of which 69 juveniles were genotyped using tube foot clips. Of the 69 *F*_1_ juveniles genotyped, 33 were identified as definitive heterozygotes by PCR and Sanger sequencing. These mature ABCB1Δ800 *F*_1_ heterozygous mutants were in-crossed (Fig. 1C) to produce *F*_2_ larvae, where we expected a Mendelian mix of homozygous (25%), heterozygous (50%) and wild-type (25%) animals (Fig. 2Aii,B). Consistent with this expectation, 32 out of 119 individual *F*_2_ larvae genotyped (Fig. S5) were wild type (26.9%), 54 were heterozygous (45.4%) and 33 were homozygous (27.7%) (Fig. 2C).

To validate these genotypes further, individual gel bands for 18 samples (six of each genotype, Fig. 2B) were extracted and sequenced directly. All six samples with a single large or a single small band were wild type or homozygous ABCB1Δ800 deletion mutants, respectively. The remaining six samples with two bands represented heterozygous ABCB1Δ800 mutants with one wild type and one deletion mutant sequence (Fig. 2Aii). We also cloned the entire PCR product for three representative homozygous samples to further examine their identities. Forty-seven clones were recovered and sequenced from these samples, of which all were ABCB1Δ800 mutants and none were wild type (Fig. S6).

### *F*_2_ ABCB1Δ800 larvae exhibit reduced transport capability

**Fig. 3.**
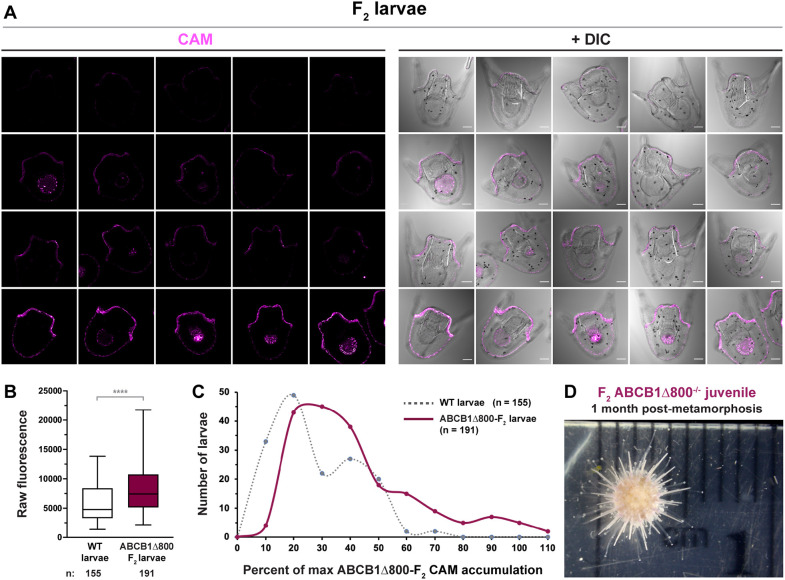
**A significant loss of transporter efflux activity is present within ABCB1Δ800 *F*_2_ line larvae.** (A,B) CAM substrate accumulation assay. ABCB1Δ800 *F*_2_ line larvae exhibit higher intracellular levels of the ABCB1 substrate CAM (magenta; A) when compared with outbred wild-type larvae (B; ****P<0.0001, unpaired two-tailed Mann–Whitney *t*-test). Micrograph examples of low, medium and maximum intracellular CAM accumulation in ABCB1Δ800 line *F*_2_ larvae are shown (A). DIC, differential interference contrast. Scale bars: 50 µm. Data are pooled from three independent mate pairs and all images were acquired and processed using the same settings. Representative wild-type images are shown in Fig. S7. (C) The distribution of CAM accumulation is broader within ABCB1Δ800 line *F*_2_ larvae. Raw fluorescence values were normalized to the maximum accumulation values. Frequency distribution of normalized values shows a shift in peak fluorescence evident in ABCB1Δ800 *F*_2_ line larvae when compared with outbred wild-type larvae. (D) Homozygous *F*_2_ ABCB1Δ800^−/−^ mutants survive metamorphosis. A representative 1-month-old juvenile is shown.

Having found a Mendelian ratio of the ABCB1Δ800 mutation in *F*_2_ larvae, we next sought to determine the range of transporter efflux activities within the mutated *F*_2_ generation by using the CAM substrate accumulation assay (Fig. 3; Fig. S7). We crossed *F*_1_ parents that were genotyped as heterozygous for the ABCB1Δ800 mutation. Their larval offspring are hereafter designated as ‘ABCB1Δ800-*F*_2_’ larvae. We also crossed parents from naturally wild-type outbred populations, the offspring of which are hereafter called ‘wild-type’ larvae. At least two distinct mate pairs were used for each type of cross and pooled for analysis.

ABCB1Δ800-*F*_2_ larvae had a significantly higher accumulation of intracellular CAM in the ectoderm when compared with wild-type larvae (Fig. 3A,B, *P*<0.0001; Fig. S7), indicating the reduction of transporter efflux activity in the ABCB1Δ800 *F*_2_ generation. The ABCB1Δ800-*F*_2_ larvae also exhibited a wider range of accumulation values (Fig. 3B), consistent with reduced efflux activity of a subset of larvae, the heterozygous (+/−) and homozygous (−/−) ABCB1Δ800 mutants, when compared with homozygous wild-type (+/+) siblings. This broad distribution of peak fluorescence values for ABCB1Δ800-*F*_2_ larvae was specifically shifted upwards when compared with outbred wild types (Fig. 3C; Fig. S7), indicating an increase in larvae with low or no ABCB1 transport activity. A total of 43 ABCB1Δ800-*F*_2_ larvae (out of 191 total, 22.5%) exhibited accumulation levels in the upper 30% of this distribution, roughly consistent with the expected Mendelian distribution of homozygous ABCB1Δ800^−/−^ *F*_2_ larvae.

When these *F*_2_ larvae were grown through metamorphosis and genotyped at the juvenile stage, ABCB1Δ800^−/−^ juveniles survived and did not exhibit any outward morphological defects (Fig. 3D). This is consistent with what has been reported in knockout mouse lines of ABCB1 (Schinkel et al., 1994, 1996).

### Conclusions

This study represents a fundamental step towards more sophisticated genetic study of the sea urchin embryo. An innovation of our approach was to bring together optimized approaches for culturing of this animal, with streamlined methods for generating and screening for mutations. Importantly, these methods can be reproduced in any lab using sea urchins. For example, in terms of culturing methods, the animals were grown in standard recirculating systems, similar to those used for zebrafish and widely available in research labs. Similarly, in terms of generation and characterization of mutants, we chose an approach that is applicable to producing a mutant of any gene that produces a non-lethal or non-visible mutation. Unlike the pigmentation gene previously targeted for stable mutagenesis in the sea urchin (Yaguchi et al., 2020), the knockout of ABCB1 does not provide a phenotype readily visible by eye in embryos or adult animals (Schinkel et al., 1994, 1996). Thus, to efficiently generate and screen for ABCB1 mutants, we used a dual guide CRISPR/Cas9 gene-editing method and generated a large genomic deletion identifiable by routine PCR (Figs 1 and 2). This diagnostic PCR is easily performed by non-destructive sampling of a small piece of tube foot.

To our knowledge, the knockout line reported here is the first ABCB1^−/−^ animal line outside vertebrates (Schinkel et al., 1994) and the second example of a homozygous knockout mutant to ever be established in sea urchins (Yaguchi et al., 2020). Most previous work on these proteins has used differentiated cell lines (Begicevic and Falasca, 2017; Robey et al., 2018). Although powerful tools, these differentiated cells do not recapitulate the membrane reorganization and cell-cell signaling events that occur during development. Importantly, ABC transporters such as ABCB1 are just a small part of the overall transporter repertoire of the embryo (Shipp and Hamdoun, 2012; Schrankel and Hamdoun, 2021), and most remain poorly understood. As such, new animal models, including *Lytechinus pictus*, could prove to be an important tool for studying small-molecule transporter functions and their regulation in embryo development.

Finally, and most broadly, a sea urchin animal resource – based on the advantageous biological features of *Lytechinus pictus* – could have many major advantages for this model organism. First, the use of lab-grown animals would reduce reliance on wild collected animals, the availability, genotype and gamete quality of which are unpredictable. Second, the generation of lines with features useful for common experiments across many labs, such as the expression of fluorescent reporters for imaging, would obviate repetitive procedures, such as mRNA injection, and thus increase efficiency across the community. Finally, these lab-grown urchins would also make the ideal building blocks for the generation of lines engineered to readily accept transgenes (e.g. animals with recombinase-based ‘targeting capabilities/landing pads’; Wirth et al., 2007). In turn, these lines would enable entirely new experiments in sea urchins, where batteries of genes and/or cis regulatory elements are controlled in a cell- and stage-specific manner. As such, genetically enabled *Lytechinus pictus* are poised to have a transformative impact on the overall reproducibility, utility and impact of research using sea urchins.

## MATERIALS AND METHODS

### *Lytechinus pictus* husbandry

#### Larval culture

Adult *L. pictus* were initially collected in San Diego, CA, USA and housed in flowing seawater aquaria at 18-22°C. Animals were spawned as previously described (Nesbit et al., 2019; Nesbit and Hamdoun, 2020). *Lp-ABCB1* crispant larvae were cultured at 22°C in glass beakers of filtered seawater (FSW) with rotating paddles and gentle aeration by a small air-stone or line. Larval cultures were fed red flagellated algae *Rhodomonas lens* at 5000-8000 algal cells per ml larval culture, starting at 2 days post fertilization (dpf). The earliest larval metamorphosis occurred between 2 and 3 weeks post-fertilization (wpf). Any remaining larvae were induced to metamorphosis at 4 wpf using KCl, as previously described (Nesbit and Hamdoun, 2020).

#### Juvenile culture

Post-metamorphic juvenile *L. pictus* were housed in 8 l plastic aquaria with sponge filters until ∼1-2 months post metamorphosis (mpm) or 2 mm test diameter (whichever occurs first). The 8 l cultures were fed with diatoms (*Nitzschia alba*; 3000-5000 cells per ml juvenile culture) every other day. A 30% water change was performed daily.

#### Sub-adult culture

Juveniles at 2 mm size were transferred to a recirculating sea water system (Aquaneering, San Diego, CA, USA) and housed individually in 1, 3 or 6 l enclosures. Juveniles during this phase of development were fed a mixture of diatoms (*Nitzschia alba*) and kelp (*Macrocystis pyrifera*) *ad libitum*. Diet was also occasionally supplemented with sea lettuce (*Ulva lactuca*) and dulce (*Palmaria palmata*), and supplemented once with some small pieces of market squid (*Doryteuthis opalescens*).

#### Adult culture

Juveniles that were 10 mm and above in size were primarily fed kelp. Spawning was induced by injecting 20-60 µl of 0.55 M KCl to collect gametes.

### Annotation of *L. pictus* ABCB1

*L. pictus* ABCB1 was initially identified by BLAST against related *S. purpuratus* ABC transporters (*Sp-ABCB1a*, *Sp-ABCB1b*, *Sp-ABCB1c* and *Sp-ABCB4a;* Warner et al., 2021). Transcripts with significant similarity (e-value <1e^−100^) were further characterized by inspection of topology and domain architecture, and aligned to the amino acid sequences of previously characterized ABCB transporters (Fig. S1A) from a variety of vertebrates and invertebrates using Geneious software (v11.1.5) (Sievers et al., 2011; Sievers and Higgins, 2018). ProtTest (v.3.4.2) was used to predict the best fit model for tree construction: A maximum likelihood tree (RaxML-HPC2 on XSEDE) with 1000 bootstraps using a LG+G model, with *Saccharomyces cerevisiae* selected as an outgroup, was created through CIPRES Science Gateway (v.3.3) (Miller et al., 2015). The phylogenetic tree was visualized using FigTree (v1.4.4), and nucleotide binding domains (NBDs), Walker A, Walker, B, Q-loop and LSQQG motifs were identified using Clustal Omega (Sievers et al., 2011; Sievers and Higgins, 2018). Topology was inferred using Topcons (Tsirigos et al., 2015) and ScanProsite (de Castro et al., 2006).

### Overexpression and experimental validation of *L. pictus ABCB1:mCherry*

The *in-silico* predicted ABCB1 ortholog was validated by overexpressing it in *L. pictus* embryos. Total RNA was isolated from the gastrula stage (24 hpf) of *L. pictus* and converted into cDNA using the SMARTer PCR cDNA synthesis kit (Takara Bio USA) according to the manufacturer's instructions. The *Lp-ABCB1* transcript was amplified using UTR to UTR primers and PrimeStar high fidelity DNA polymerase (Takara Bio USA). Primers used in this study are listed in Table S1. The open reading frame was subcloned into the PCS2+8 NmCherry plasmid using In-Fusion cloning (Takara Bio USA) and *in vitro* transcription was performed as previously described (Gökirmak et al., 2012, 2014) using the mMessage mMachine kit (Thermo Fisher Scientific). Zygotes were microinjected with 250 ng/µl of *Lp-ABCB1:NmCherry* mRNA or 50 ng/µl of the histone marker *H2B:NmCherry* mRNA as a control (Shipp et al., 2015).

### *Lp-ABCB1* CRISPR/Cas9 guides design and microinjection

ChopChop v.2 (https://chopchop.cbu.uib.no) (Labun et al., 2016) was used to design single guide RNAs (sgRNAs) to target *Lp-ABCB1* as previously described (Fleming et al., 2021). To create a loss-of-function mutation in *Lp-ABCB1*, target sites were identified before the 1st NBD, which is crucial for ATPase activity and efflux. The first cytoplasmic domain (located between MSD1 and MSD2, and containing the 1st NBD) of ABCB1 begins in exon 10, with the 1st NBD starting at exon 11. One target site in each exon was identified, exon 10+76 (Ex10+76) and exon 11−152 (Ex11-152); + and − indicates orientation relative to the ORF, and the number represents the base adjacent to the PAM site in the orientation of the ORF. These sgRNAs candidates have low predicted off-target sites based on BLAST analyses against the *L. pictus* genome and transcriptome (Warner et al., 2021; Table S2). Synthetic sgRNAs (Ex10+76, UGGAGACUUCACCACGGCCA; Ex11-152, GCUGCUGCCGACCAUGGCGA) were ordered from Synthego. The sgRNAs were rehydrated in nuclease-free water to 3300 ng/µl, diluted to 900 ng/µl aliquots and stored at −80°C for further use. Injections with 750 ng/µl of Cas9 mRNA and 150 ng/µl of each sgRNA were performed to create mutations simultaneously at both target sites. A positive control injection membrane marker LCK:mCherry mRNA (25 ng/µl) was co-injected. A total of 235 and 280 zygotes from two independent mate pairs were injected and raised as *F*_0_ *Lp-ABCB1* crispants.

### Genotyping of *Lp-ABCB1* mutant lines

#### Genomic DNA extraction

*Lp-ABCB1* crispants (*F*_0_) and all of their future progeny (*F*_1_ and *F*_2_) were genotyped at different developmental stages to verify the presence of indels at the exon 10/11 target sites. Genomic DNA was isolated using the Purelink Genomic DNA mini kit (Thermo Fisher Scientific) or the QIAamp DNA micro kit (Qiagen) according to the manufacturers' instructions. All genomic DNA samples were eluted in 50 µl elution buffers and stored at −20°C. For larval genotyping, a single larva or a batch of pooled 50 larvae were used for genomic extraction. At juvenile stages, two or three tube feet per juvenile were used for genomic extraction. At adult stage, genomic DNA extraction was carried out from gametes.

#### Identification of mutant alleles

The ∼3 kb region between exon 8 and exon 12 of *Lp-ABCB1* was amplified with PrimeStar high-fidelity DNA polymerase (Takara Bio USA), followed by a nested PCR with exon 9 and exon 11 specific primers (Table S1). For all genomic samples from the *F*_0_ generation, the target region was cloned into pMiniT 2.0 using the NEB PCR cloning kit (NEB) and individual colonies were Sanger sequenced to identify different types of mutations present. All genomic samples from the *F*_1_ generation with large deletions were directly sequenced from PCR fragments. Genomic samples from the *F*_2_ generation were directly sequenced from PCR fragments and cloned for additional validation. Identification of mutant alleles and alignments were generated using Sequencher (v.5.0.1) and Snapgene (5.1.4.1) alignment software.

### Lp-ABCB1 transporter efflux activity assays

Lp-ABCB1 transporter activity in wild-type, overexpressed and crispant *F*_0_ and *F*_2_ larvae was assessed by quantifying the accumulation of calcein acetoxymethyl ester (CAM; eBioscience, Thermo Fisher Scientific), as previously described (Gökirmak et al., 2012; Fleming et al., 2021). All stocks were prepared such that the final DMSO concentration did not exceed 0.5%. Efflux assays were conducted at the blastula stage (5 hpf; *Lp-ABCB1:mCherry* overexpression) or at the gastrula and larval stages (24 and 44-48 hpf; *Lp-ABCB1* crispants at *F*_0_ and *F*_2_ generations, respectively). Injected and control uninjected embryos were incubated with CAM (250 nM) in FSW for 60 min and immediately imaged. Embryos were imaged on a Zeiss LSM 700 confocal microscope using a 20× Plan-Apo, 0.8 NA air objective. All samples were imaged with identical confocal settings (pinhole size, gain, laser power, zoom and scan speed). Measurements were made from single-plane, equatorial confocal sections of embryos or larvae in which the ectoderm was in maximum focus. Image analysis was performed using ImageJ software (Schindelin et al., 2012) to measure the intracellular substrate fluorescence intensity per pixel in the ectoderm. The relative amount of substrate accumulation within tissues is used as a proxy for transporter activity.

For *Lp-ABCB1:mCherry* and *H2B:mCherry* overexpression experiments, individual fluorescence measurements of CAM were normalized to the average wild-type fluorescence of each mate pair, and the average percent change compared with wild-type control was calculated for each treatment. Statistics were performed using JMP statistical software (JMP Pro v15, SAS). For CRISPR/Cas9 editing validation, individual fluorescence measurements of CAM in crispant *F*_0_ larvae were compared with the average fluorescence of wild-type controls for each mate pair.

For assessing the range of accumulation phenotypes in ABCB1Δ800 *F*_2_ larvae, individual fluorescence measurements were normalized to the average of the top five maximum accumulation values in ABCB1Δ800 *F*_2_ larvae. Statistics and frequency distribution analysis were performed using Prism GraphPad statistical software (version 9.1.2).

## Supplementary Material

Supplementary information
