# Supplementary material for: Generation of a homozygous mutant drug transporter (ABCB1) knockout line in the sea urchin Lytechinus pictus
Source: Development. 2022 Jun 6;149(11):dev200644. doi: 10.1242/dev.200644 (PMC9245184; doi:10.1242/dev.200644)
Supplement: Supplementary information [file develop-149-200644-s1.pdf]

SUPPLEMENTARY INFORMATION

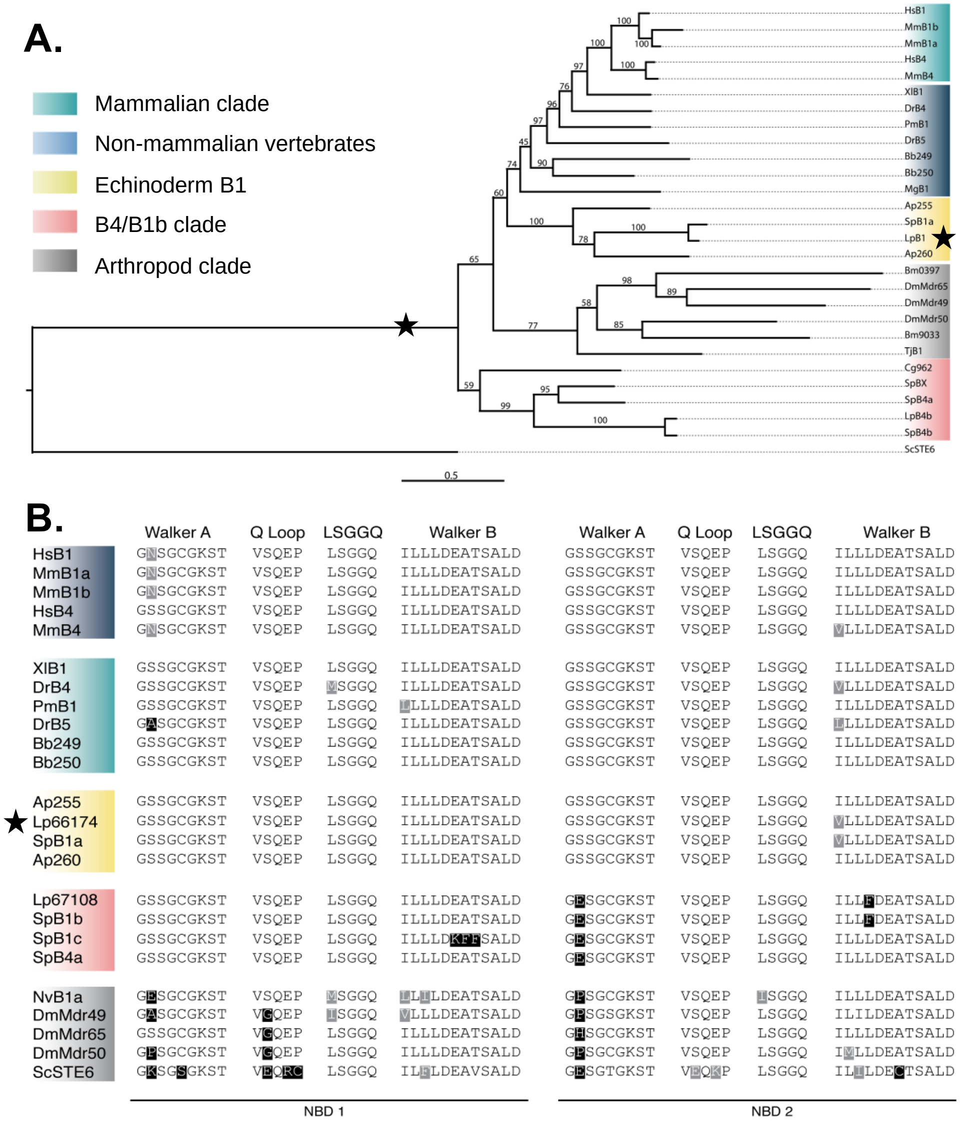

Fig. S1. Identification of the *Lp-ABCB1* gene.

A. Phylogenetic tree of ABCB1 homologs. Nucleotide binding domain (NBD) amino acid sequences were used to construct a maximum likelihood tree of ABCB1 homologs. Genes are listed as a two-letter abbreviation for the species, followed by the gene name or gene model ID (e.g., LpB1 for *Lytechinus pictus* ABCB1, starred). Sea urchin ABCB1a genes are more similar to chordate ABCB transporters, whereas echinoderm ABCB4/ABCB1b/ABCB1c are more similar to protostome and non-bilaterian ABCB

Development • Supplementary information

transporters. Groupings of major clades are color coded: mammalian B1 (green), other vertebrate B1 and zebrafish B4 (blue), echinoderm B1 (yellow), arthropods (grey), and echinoderm B4 paralogs (red). Yeast (*Saccharomyces cerevisiae*) serves as the outgroup for the tree. Bootstraps (1000) are shown for each branch. Ap (*Acanthaster planci*), Bb (*Branchiostoma belcheri*), Bm (*Bombyx mori*), Cg (*Crassostrea gigas*), Dm (*Drosophila melanogaster*), Dr (*Danio rerio*), Hs (*Homo sapiens*), Lp (*Lytechinus pictus*), Mg (*Mytilus galloprovincialis*), Mm (*Mus musculus*), Pm (*Petromyzon marinus*), Sc (*Saccharomyces cerevisiae*), Sp (*Strongylocentrotus purpuratus*), Tj (*Tigriopus japonica*), Xl (*Xenopus laevis*). **B. NBD motifs differ between chordate ABCB clades and the echinoderm ABCB4, protostome, and non-bilaterian ABCB clades.** The Walker A site in the NBD2 of the ABCB1 clades contains a serine at residue two, while the echinoderm ABCB4a and protostome and non-bilaterian ABCB clades do not have a fixed residue at that position (boxed in black). This suggests that while the last common ancestor of the deuterostomes had two P-gp like ABCB transporters, only one of these genes gave rise to the pair of chordate P-gp-like ABCB transporters seen in most vertebrates. Further assembly of high-quality invertebrate deuterostome genomes may allow us to elucidate this transition more accurately.

Development • Supplementary information

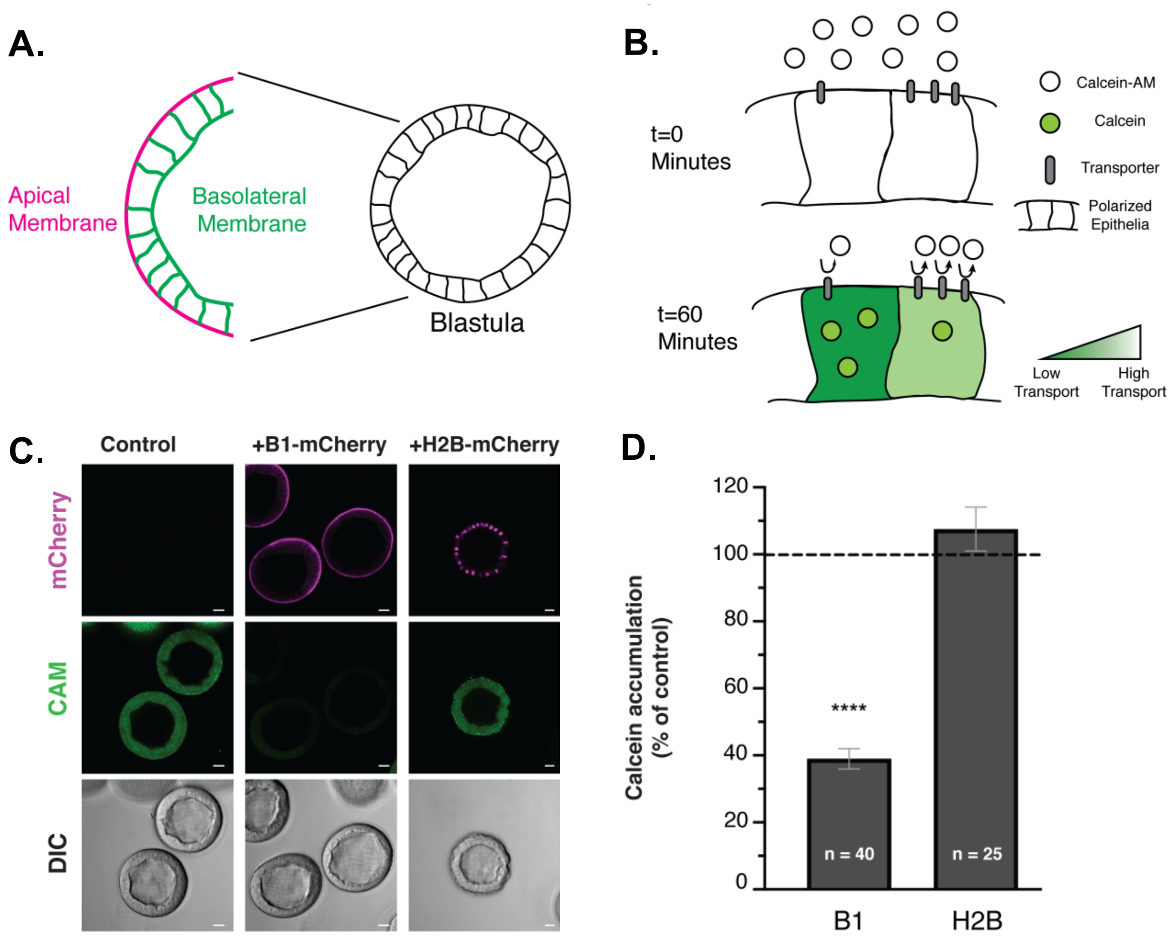

Fig. S2. Validation of *Lp-ABCB1.*

A. Schematic of sub-cellular localization in a sea urchin blastula. Overexpression of fluorescent tagged ABCB1 is expected in the apical membrane (magenta). **B. The Calcein-AM (CAM) assay for quantifying transporter activity.** Once inside the cell, CAM becomes cleaved into a fluorescent calcein that cannot passively exit the cell. Cells with high transporter activity have reduced accumulation of fluorescent calcein compared to cells with low transporter activity. **C. Lp-ABCB1 effluxes calcein from epithelia.** Lp-ABCB1:mCherry (magenta) localizes to the apical membrane and over-expressing embryos accumulate significantly less calcein (green) than control or histone H2B:mCherry expressing embryos. Scale bars, 25 µm. **D. Quantification of the CAM assay.** ****, $p < 0.0001$ (unpaired two-tailed Mann-Whitney t-test). Data are pooled from 4 independent mate pairs. Error bars represent standard deviation.

Development • Supplementary information

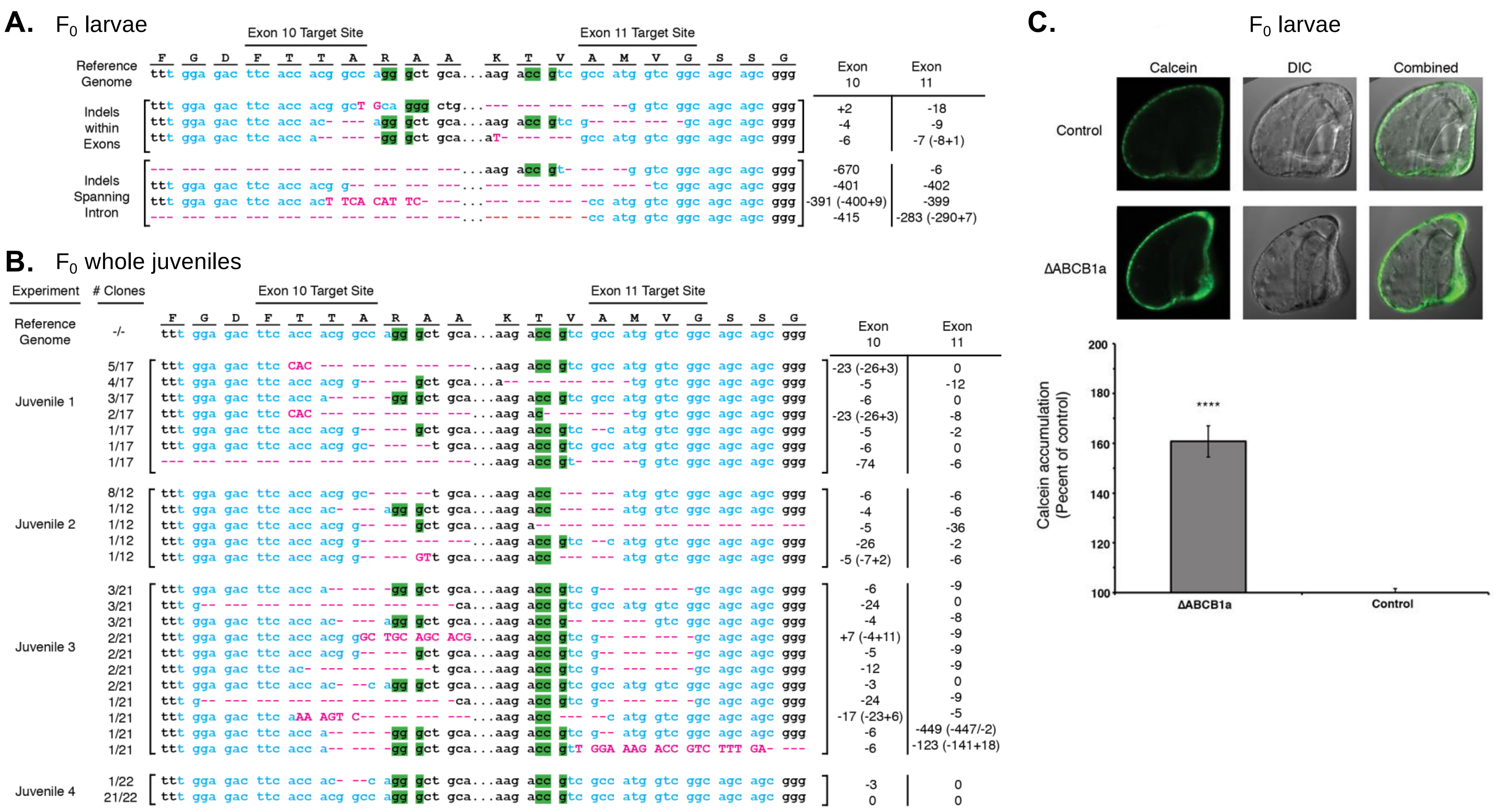
A. F0 larvae
Exon 10 Target Site
Exon 11 Target Site
F G D F T T A R A A K T V A M V G S S G
Reference Genome
ttt gga gac ttc acc acg gcc agg gct gca...aag acc gtc gcc atg gtc ggc agc agc ggg
Exon 10
Exon 11
Indels within Exons
ttt gga gac ttc acc acg gcT Gca ggg ctg...--- --- --- --- --g gtc ggc agc agc ggg +2 -18
ttt gga gac ttc acc ac- --- agg gct gca...aag acc gtc g-- --- --- -gc agc agc ggg -4 -9
ttt gga gac ttc acc a-- --- -gg gct gca...aT- --- --- gcc atg gtc ggc agc agc ggg -6 -7 (-8+1)
Indels Spanning Intron
--- --- --- --- --- --- --- --- --- ---...aag acc gt- --- --g gtc ggc agc agc ggg -670 -6
ttt gga gac ttc acc acg g-- --- --- ---...--- --- --- --- ---- -tc ggc agc agc ggg -401 -402
ttt gga gac ttc acc acT TCA CAT TC- ---...--- --- --- -cc atg gtc ggc agc agc ggg -391 (-400+9) -399
--- --- --- --- --- --- --- --- --- ---...--- --- --- -cc atg gtc ggc agc agc ggg -415 -283 (-290+7)
B. F0 whole juveniles
Experiment
Clones
Exon 10 Target Site
Exon 11 Target Site
F G D F T T A R A A K T V A M V G S S G
Reference Genome -/- ttt gga gac ttc acc acg gcc agg gct gca...aag acc gtc gcc atg gtc ggc agc agc ggg
Exon 10
Exon 11
Juvenile 1
5/17 ttt gga gac ttc CAC --- --- --- --- ---...aag acc gtc gcc atg gtc ggc agc agc ggg -23 (-26+3) 0
4/17 ttt gga gac ttc acc acg g-- --- gct gca...a-- --- --- --- -tg gtc ggc agc agc ggg -5 -12
3/17 ttt gga gac ttc acc a-- --- -gg gct gca...aag acc gtc gcc atg gtc ggc agc agc ggg -6 0
2/17 ttt gga gac ttc CAC --- --- --- --- ---...aag ac- --- --- -tg gtc ggc agc agc ggg -23 (-26+3) -8
1/17 ttt gga gac ttc acc acg g-- --- gct gca...aag acc gtc --c atg gtc ggc agc agc ggg -5 -2
1/17 ttt gga gac ttc acc acg gc- --- --t gca...aag acc gtc gcc atg gtc ggc agc agc ggg -6 0
1/17 --- --- --- --- --- --- --- --- --- ---...aag acc gt- --- --g gtc ggc agc agc ggg -74 -6
Juvenile 2
8/12 ttt gga gac ttc acc acg gc- --- --t gca...aag acc --- --- atg gtc ggc agc agc ggg -6 -6
1/12 ttt gga gac ttc acc ac- --- agg gct gca...aag acc --- --- atg gtc ggc agc agc ggg -4 -6
1/12 ttt gga gac ttc acc acg g-- --- gct gca...aag a-- --- --- --- --- --- --- --- --- -5 -36
1/12 ttt gga gac ttc acc acg g-- --- --- ---...aag acc gtc --c atg gtc ggc agc agc ggg -26 -2
1/12 ttt gga gac ttc acc acg g-- --- GTt gca...aag acc --- --- atg gtc ggc agc agc ggg -5 (-7+2) -6
Juvenile 3
3/21 ttt gga gac ttc acc a-- --- -gg gct gca...aag acc gtc g-- --- --- -gc agc agc ggg -6 -9
3/21 ttt g-- --- --- --- --- --- --- --- -ca...aag acc gtc gcc atg gtc ggc agc agc ggg -24 0
3/21 ttt gga gac ttc acc ac- --- agg gct gca...aag acc g-- --- --- gtc ggc agc agc ggg -4 -8
2/21 ttt gga gac ttc acc acg gGC TGC AGC ACG...aag acc gtc g-- --- --- -gc agc agc ggg +7 (-4+11) -9
2/21 ttt gga gac ttc acc acg g-- --- gct gca...aag acc gtc g-- --- --- -gc agc agc ggg -5 -9
2/21 ttt gga gac ttc ac- --- --- --- --t gca...aag acc gtc g-- --- --- -gc agc agc ggg -12 -9
2/21 ttt gga gac ttc acc ac- --c agg gct gca...aag acc gtc gcc atg gtc ggc agc agc ggg -3 0
1/21 ttt g-- --- --- --- --- --- --- --- -ca...aag acc gtc g-- --- --- -gc agc agc ggg -24 -9
1/21 ttt gga gac ttc aAA AGT C-- --- --- ---...aag acc --- --c atg gtc ggc agc agc ggg -17 (-23+6) -5
1/21 ttt gga gac ttc acc a-- --- -gg gct gca...aag acc gtc g-- atg gtc ggc agc agc ggg -6 -449 (-447/-2)
1/21 ttt gga gac ttc acc a-- --- -gg gct gca...aag acc gtT GGA AAG ACC GTC TTT GA- --- -6 -123 (-141+18)
Juvenile 4
1/22 ttt gga gac ttc acc ac- --c agg gct gca...aag acc gtc gcc atg gtc ggc agc agc ggg -3 0
21/22 ttt gga gac ttc acc acg gcc agg gct gca...aag acc gtc gcc atg gtc ggc agc agc ggg 0 0
C.
F0 larvae
Calcein
DIC
Combined
Control
ΔABCB1a
Calcein accumulation (Pecent of control)
200
180
160
140
120
100
****
ΔABCB1a
Control

Fig. S3. Identification and validation of mutations in F_0 crispants.

A. The F_0 generation exhibits several types of indels and a loss of transporter activity. Genomic DNA was extracted, amplified and cloned from pooled larvae of the F_0 generation (**A**). Blue, sgRNA target sites. Green, PAM site. Magenta, indels. Total sizes of indels are indicated in the table. **B. Mosaic integration of ABCB1Δ indels cloned from whole F_0 juveniles.** Sequence information is shown as in (**A**). **C. ABCB1Δ F_0 larvae exhibit increased accumulation of the ABCB1 substrate CAM.** Images are representative of n=67 WT and n=57 ABCB1Δ F_0 larvae pooled from 7 separate F_0 batches; ****, p =7.742E-06, (unpaired two-tailed t-test). Error bars represent standard deviation. DIC, differential interference contrast.

Development • Supplementary information

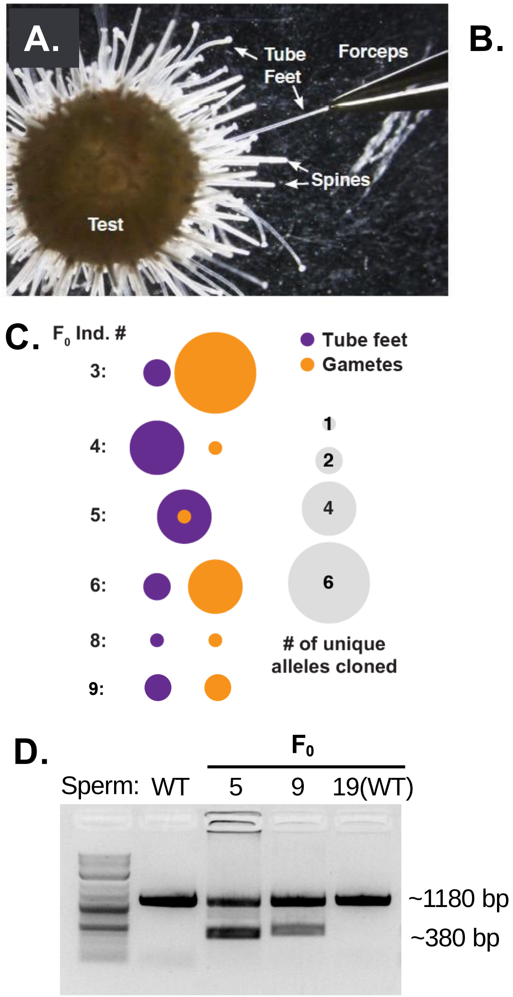

B.

Individual	Samples	# Clones	Exon 10 Target Site / Exon 11 Target Site (F G D F T T A R A A … K T V A M V G S S G)	Exon 10	Exon 11
Reference Genome	n/a	n/a	ttt gga gac ttc acc acg gcc agg gct gca...aag acc gtc gcc atg gtc ggc agc agc ggg		
#3	Tube feet	3/13	ttt gga gac t-- --- --- --- --- --- ---...--- --- -tc gcc atg gtc ggc agc agc ggg	-53	-151
		10/13	ttt gga gac ttc acc acA CCT GAT GTG CAA...aag acc gtc g-- --- --- -gc agc agc ggg	+28	-9
	Sperm	18/30	ttt gga gac ttc acc ac- --- agg gct gca...aag acc gtc gGC AGC AGt ggt cgg cag cag	-4	+4 (-3+7)
		3/30	ttt gga gac ttc acc ac- --- agg gct gca...aag acc gtc --- --- --- --- agc agc ggg	-4	-12
		2/30	ttt gga gac ttc acc acT TCA Cca ggg ctg...aag acc gtc gGC AGC AGt ggt cgg cag cag	+2 (-3+5)	+4 (-3+7)
		6/30	ttt gga gac ttc acc acT TCA Cca ggg ctg...aag acc gtc --- --- --- --- agc agc ggg	+2 (-3+5)	-12
		1/30	ttt gga gac ttc acc ac- --- TTg gct gca...aag acc gtc gGC AGC AGt ggt cgg cag cag	-4 (-6+2)	+4 (-3+7)
#4	Tube feet	4/10	ttt gga gac ttc acc ac- --- --- --- ---...aag acc gtc g-- --- --- -gc agc agc ggg	-660	-9
		3/10	ttt gga gac ttc acc acg g-- --- gct gca...--- --- --- --- --- --- -gc agc agc ggg	-5	-18 (-26+8)
		2/10	ttt gga gac ttc acc a-- --- agg gct gca...aag acc gtc gc- atg gtc ggc agc agc ggg	-5	-1
		1/10	ttt gga gac ttc acc a-- --- agg gct gca...--- --- --- --- --- --- -gc agc agc ggg	-5	-18 (-26+8)
	Sperm	50/50	ttt gga gac ttc acc acg g-- --- gct gca...aag acc gtc -cc atg gtc ggc agc agc ggg	-5	-1
#5	Tube feet	3/10	ttt gga gac ttc acc acg --- --- --- ---...--- --- --- --- --- --c ggc agc agc ggg	-345	-460
		3/10	ttt gga gac ttc acc acg g-- --- --- ---...--- --- --- --- --- -tg gtc ggc agc agc ggg	-344	-456
		2/10	ttt gga gac ttc --- --- --- --- --- ---...aag acc gtc gcc atg gtc ggc agc agc ggg	-103	-0
		2/10	--- --- --- --- --- --g gcc agg gct gca...aag acc gtc gcc atg gtc ggc agc agc ggg	+21 (+61-40)	-0
	Sperm	17/17	ttt gga gac ttc acc acg g-- --- --- ---...--- --- --- --- --- -tg gtc ggc agc agc ggg	-344	-456
#6	Tube feet	14/16	ttt gga gac ttc acc ac- --c agg gct gca...aag acc gtc g-- --- --- -gc agc agc ggg	-3	-9
		2/16	ttt gga gac ttc acc acT TCA Cca ggg ctg...aag acc gtc gcc atg gtc ggc agc agc ggg	+2 (-3+5)	0
	Sperm	17/20	ttt gga gac ttc acc acg gc- --- --t gca...aag acc gtc gcc Cat ggt cgg cag cag cgg	-6	+1
		1/20	ttt gga gac ttc acc acg gc- --- --t gca...--- --- --- --- --- gtc ggc agc agc ggg	-6	-21
		1/20	ttt gga gac ttc acc acg g-- --- --- ---...--- --- --- --- -tg gtc ggc agc agc ggg	-344	-456
		1/20	ttt gga gac ttc acc acT TCA Cca ggg ctg...aag acc gtc gcc Cat ggt cgg cag cag cgg	+2 (-3+5)	-+1
#8	Tube feet	1/13	ttt gga gac ttc acc acT TCA CAT TC- ---...--- --- --- -cc atg gtc ggc agc agc ggg	-391(-400+9)	-399
		12/13	ttt gga gac ttc acc acg gcc agg gct gca...aag acc gtc gcc atg gtc ggc agc agc ggg	0	0
	Eggs	1/37	ttt gga gac ttc acc acg gcc agg gct gca...aag acc gtc gGC AGC AGt ggt cgg cag cag	0	+4 (-7+3)
		36/37	ttt gga gac ttc acc acg gcc agg gct gca...aag acc gtc gcc atg gtc ggc agc agc ggg	0	0
#9	Tube feet	12/13	ttt gga gac ttc acc a-- --- -gg gct gca...aag acc --- --- atg gtc ggc agc agc ggg	-6	-6
		1/13	ttt gga gac ttc acc acg gcc GGC aCg gct...aag acc --- --- atg gtc ggc agc agc ggg	+4	-6
	Sperm		ttt gga gac ttc acc acg gTc TGc agg gct...aag acc gtc g-- --- --- -gc agc agc ggg	+3	-9
			--- --- --- --- --- --- --- --- --- ---...aag acc gtc gcc atg gtc ggc agc agc ggg	-711	0

Fig. S4. Identification of mosaicism in mutant founder juveniles.

A. Non-invasive tube foot clipping for genotyping. Tube feet are safely removed from individual juveniles greater than 3 mm in test diameter (starting between 3-4 months post metamorphosis). Like tail clips in mice or fin clips in zebrafish, this method is non-lethal. Sea urchin tube feet regenerate and up to five tube feet can be removed at a time. **B-C. ABCB1Δ indels cloned from F_0 juveniles differ between somatic and germline tissues.** Genomic DNA was extracted, amplified and cloned from tube feet and gametes of the F_0 generation to identify founder animals (**B**). Blue, sgRNA target sites. Green, PAM site. Magenta, indels. Total sizes of indels are indicated in the table to the right. Animal #5 (boxed in maroon) had a single large deletion mutation (hereafter designated ABCB1Δ800) present in sperm and was selected for future crosses. Aside from animal #5, mutations present in somatic tissue were rarely found in the germline (**B-C**). **D. PCR screening of F_0 ABCB1Δ sperm.** Large deletion mutant alleles were detected in samples from F_0 #5 and #9, while somatic wild type F_0 #19 showed only WT alleles, identified by gel band pattern.

Development • Supplementary information

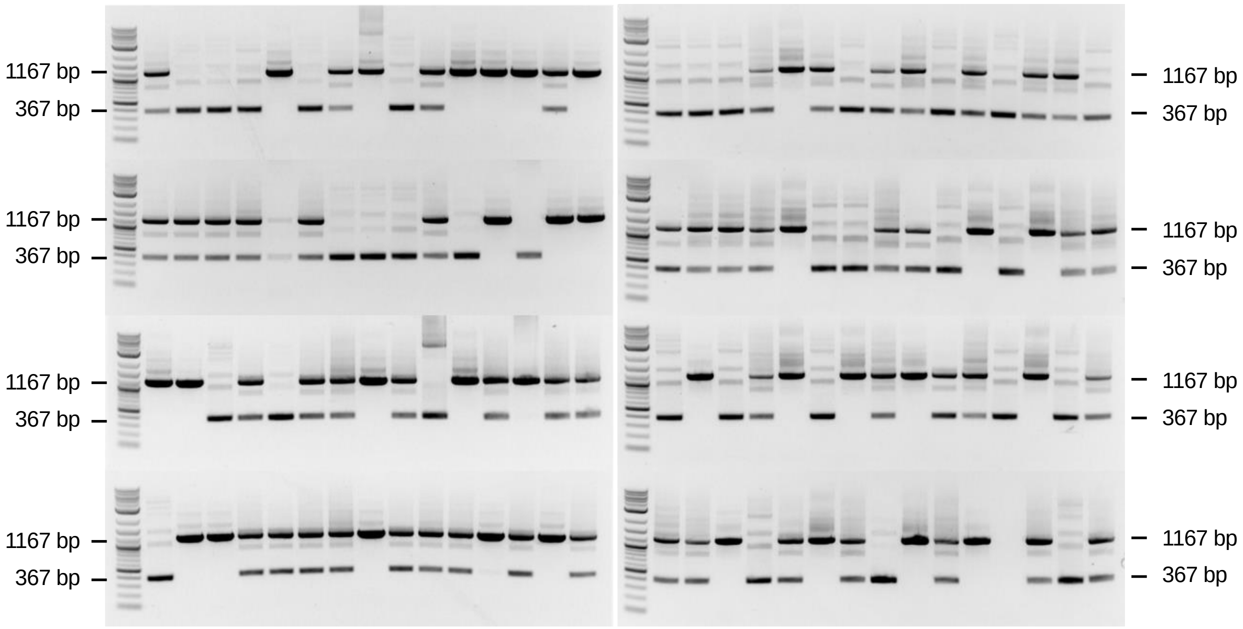

Fig. S5. Verification of ABCB1Δ800 F_2 larvae genotypes

Individual larvae (n=120) were genotyped by PCR at the *Lp-ABCB1* locus. WT (**+/+**, 1167 bp), heterozygous (**+/-**, 1167/367 bp) and homozygous mutants (**-/-**, 367 bp) are identified by gel band pattern. To further verify the genotypes, individual gel bands for 18 samples (six of each genotype, shown in Fig. 2B) were purified and sequenced directly. All six samples with a single large or a single small band had WT or Homozygous ABCB1Δ800 deletion sequence, respectively. The remaining six samples with two bands (representing Heterozygous ABCB1Δ800 mutants) generated one WT and one deletion mutant sequence. Three representative homozygous samples were also sequenced directly from PCR products. WT sequence was not found in the sequenced clones from these samples (n=47; see Fig. S6).

Development • Supplementary information

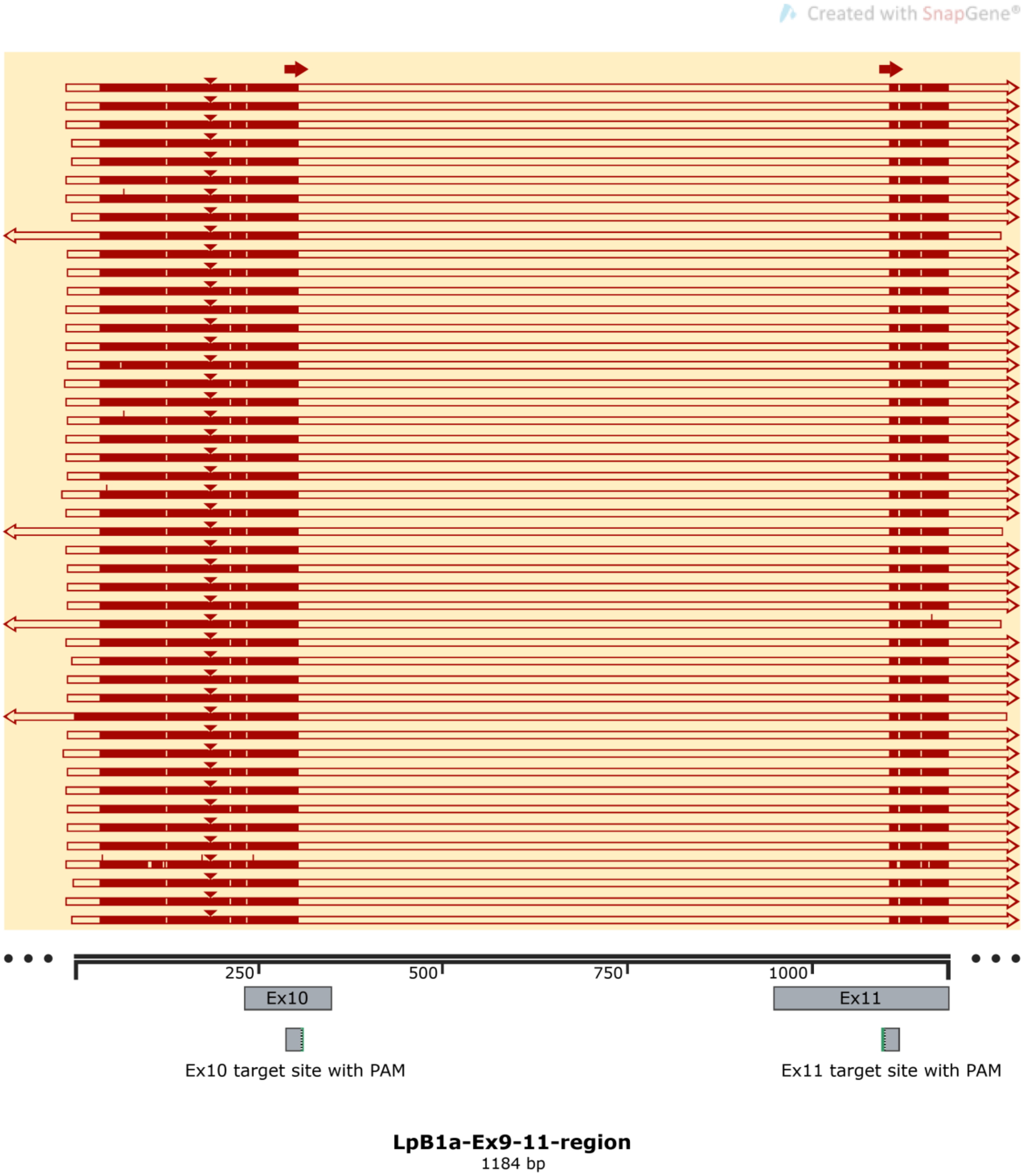

Fig. S6. Validation of ABCB1Δ800 homozygous F_2 larvae by sequencing clones.

Three representative homozygous samples were cloned and sequenced. Each red bar represents a single clone. Small red arrows at the top mark the target sites in Ex10 and Ex11. A total of 47 clones were sequenced across all three samples. WT sequence was not found in the sequenced clones from these samples.

Development • Supplementary information

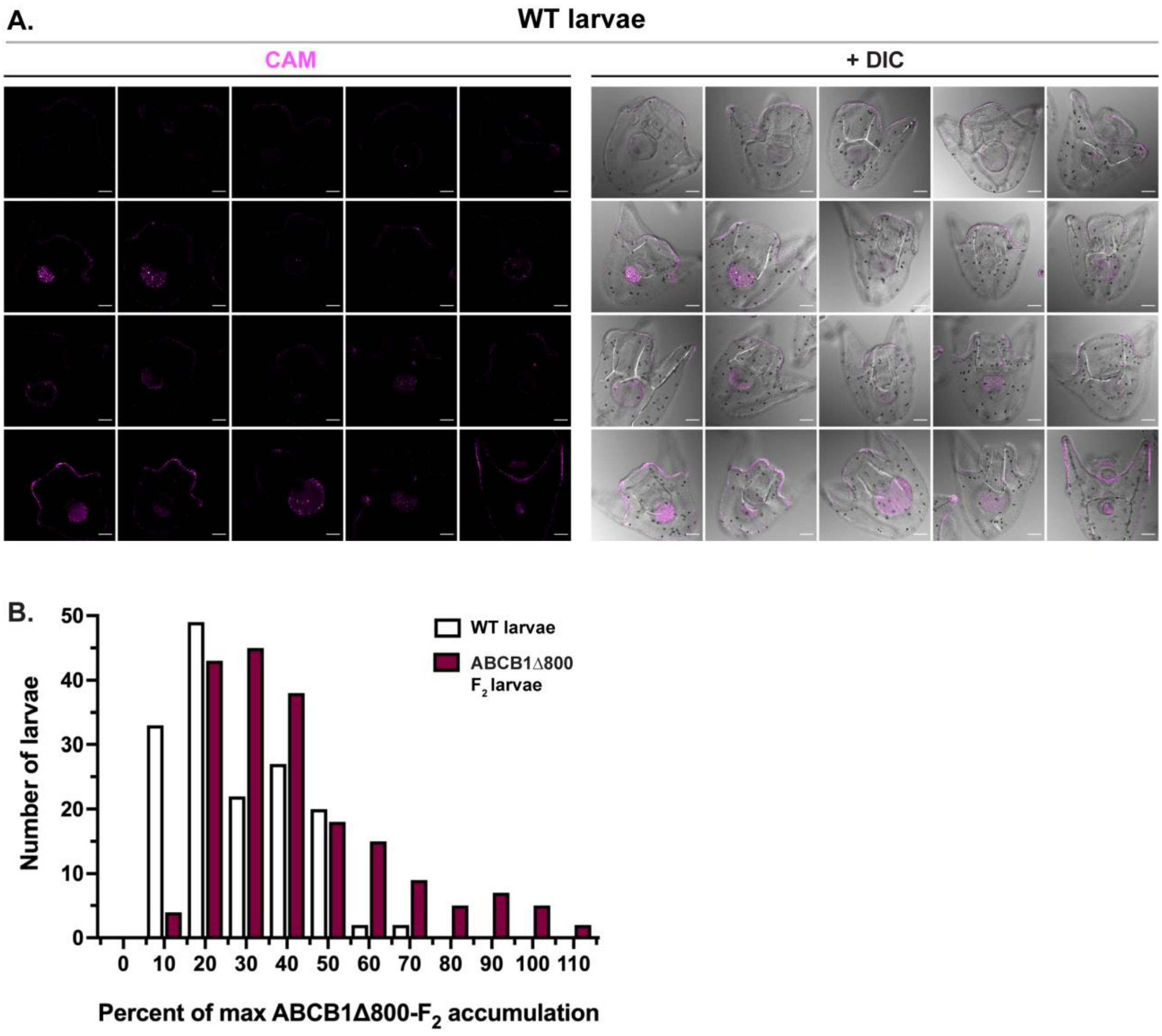

Fig. S7. ABCB1Δ800 F_2 larvae exhibit a range of increased CAM accumulation phenotypes compared to outbred WT larvae.

A. Intracellular CAM accumulation in WT larvae. Micrographs of CAM (magenta) accumulation are representative from three independent crosses of WT animals. All images were acquired and processed using the same settings. DIC, differential interference contrast. Scale bars, 50 µm. **B. Histogram of CAM accumulation phenotypes.** Raw fluorescent values were normalized to the maximum accumulation values observed in ABCB1Δ800 F_2 larvae and plotted for frequency distribution.

Table S1. Primers used in this study.

Primer Name	Sequence (5'-3')
LpB1-5utr-1	ccacgttattatctgcggcacc
LpB1-3utr-1	ctgggaaccaatctatgcggttcttt
Ex8-Forward2	cctattcttctcaatgttctccagctatgc
Ex12-Reverse2	ctctggaagcttggagatgaagtcatg
LpB1-Ex9-m13Forward	tgtaaaacgacggccagtacggcacagttctttatttagatggtga
LpB1-Ex11-m13Reverse	caggaaacagctatgacgataataatgatcatgatggtagtaatgatgac
LpB1_E8-I8F	tgccatcgccttctggtgagaacactgcaaaca
LpB1_E9-I9F	ctggagacattcttacggtaagtcaacaact
LpB1_I12-E12R	caccattcctggagcttacctctggaagcttgga
LpB1_I11-E11R	cattctaatcaggtacttacgcttccatcagca
Lp-ABCB1-Ex11-R	cgcttccatcagcaacatcatagaac

Table S2. gRNA alignments to most similar targets in the *Lytechinus pictus* genome.

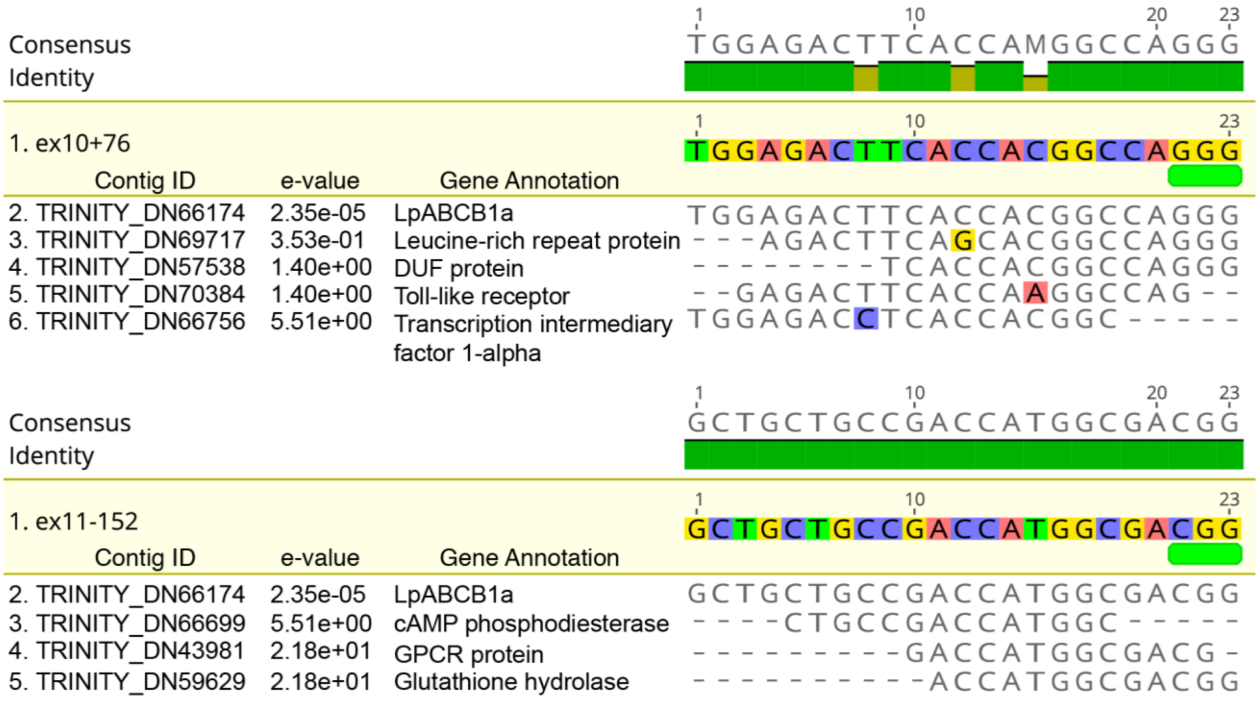

	Contig ID	e-value	Gene Annotation	Alignment
Consensus				TGGAGACTTCACCAMGGCCAGGG
1. ex10+76				TGGAGACTTCACCACGGCCAGGG
2.	TRINITY_DN66174	2.35e-05	LpABCB1a	TGGAGACTTCACCACGGCCAGGG
3.	TRINITY_DN69717	3.53e-01	Leucine-rich repeat protein	---AGACTTCAGCACGGCCAGGG
4.	TRINITY_DN57538	1.40e+00	DUF protein	--------TCACCACGGCCAGGG
5.	TRINITY_DN70384	1.40e+00	Toll-like receptor	--GAGACTTCACCAAGGCCAG--
6.	TRINITY_DN66756	5.51e+00	Transcription intermediary factor 1-alpha	TGGAGACCTCACCACGGC-----

	Contig ID	e-value	Gene Annotation	Alignment
Consensus				GCTGCTGCCGACCATGGCGACGG
1. ex11-152				GCTGCTGCCGACCATGGCGACGG
2.	TRINITY_DN66174	2.35e-05	LpABCB1a	GCTGCTGCCGACCATGGCGACGG
3.	TRINITY_DN66699	5.51e+00	cAMP phosphodiesterase	----CTGCCGACCATGGC-----
4.	TRINITY_DN43981	2.18e+01	GPCR protein	---------GACCATGGCGACG-
5.	TRINITY_DN59629	2.18e+01	Glutathione hydrolase	----------ACCATGGCGACGG

BLASTn analysis of gRNAs (PAM sites underlined in light green) against the *Lytechinus pictus* transcriptome. BLASTn was performed in Geneious v11.1.5 using the following parameters: max e-value 1000, word size 7, scoring mismatch 1-3, and gap cost 5 2. Results are presented as a query-centric alignment to contigs in the transcriptome with significant similarity. Mismatches in nucleotides are colored within the alignment. Dashes represent gaps.

Table S3. Full genotypes of Lp-ABCB1 F_0 juveniles

Individual	Variant	Copies	Exon 10	Exon 11	Total Change	Mutation Type
1	1	11	-394	0	-394	FS
	2	1	-394	-1	-395	FS
	3	1	-394	-21	-415	FS
	4	1	-5	0	-5	FS
	5	1	-5	-9	-14	FS
2	1	8	-5	-9	-14	FS
	2	4	-5	-3	-8	FS
	3	1	+1 (+4-3)	-9	-8	FS
	4	1	-5 (+1-6)	-9	-14	FS
	5	1	-5	0	-5	FS
3	1	10	+28 (+30-2)	-9	+19	FS
	2	3	-353 (+1-354)	-450	-803	FS
4	1	4	-660	-9	-669	FS
	2	3	-5	-18 (+8-26)	-23	FS
	3	2	-5	-1	-6	FS
	4	1	-5	-18 (+8-26)	-23	FS
	5	1	-345	0	-6	NFS
5	1	3	-345	-460	-805	FS
	2	3	-344	-456	-800	FS
	3	2	-103	0	-103	FS
	4	2	+21 (+61-40)	0	+21	NFS
6	1	14	-3	-9	-12	NFS
	2	2	+2 (+5-3)	0	2	FS
7	1	14	-6	0	-6	NFS
	2	1	-6	0	-6	NFS
	3	1	-5	0	-5	FS
	4	1	-344	-459	-803	FS
8	1	17	0	0	0	WT
	2	1	-338 (+9-347)	-453	-791	FS
9	1	12	-6	-6	-12	NFS
	2	1	+40	-6	+34	FS
10	1	10	-6	0	-6	NFS
	2	3	-18	0	-18	NFS
	3	1	-18	0	-18	NFS
	4	1	-3	0	-3	NFS
11	1	16	0	0	0	WT
12	1	15	0	0	0	WT

13	1	16	0	0	0	WT
14	1	10	0	0	0	WT
15	1	10	0	0	0	WT
16	1	2	-5	0	-5	FS
	2	6	0	0	0	WT
17	1	12	-229 (+12-241)	-28	-257	FS
	2	10	-6	-6 (+15-21)	-12	NFS
	3	1	-6	-28	-34	FS
	4	1	-5	-9	-14	FS
	5	1	+28 (+30-2)	-9	+19	FS
	6	2	0	0	0	WT
18	1	2	-229 (+12-241)	-28	-257	FS
	2	1	+28 (+30-2)	-9	+19	FS
	3	1	-395	0	-395	FS
	4	1	-6	-6 (+15-21)	-12	NFS
	5	3	0	0	0	WT
19	1	8	0	0	0	WT

Each juvenile was genotyped by cloning and sequencing the target locus. “Variants” represent different types of genomic sequences identified across all clones. “# clones” shows the number of clones identified for each type of variant. “Indel at Ex10” and “Indel at Ex11” show the number of bases deleted or inserted at each exon. “Mutation type” characterizes the mutation depending on the “Total change” in the genomic sequence of each variant. FS, Frameshift mutation; NFS, Non-frameshift mutation; WT, Wild type.

Development • Supplementary information
